# What accounts for multifinality of the pathways from family ecological adversity to children’s future antisocial outcomes? Exploring early attachment relationships as a source of resilience in low- and high-risk samples

**DOI:** 10.1017/S0954579425100904

**Published:** 2025-11-13

**Authors:** Juyoung Kim, Haley M. Herbert, Grazyna Kochanska

**Affiliations:** Department of Psychological and Brain Sciences, The University of Iowa, Iowa City, IA, USA

**Keywords:** Antisocial outcomes, attachment security, ecological adversity, longitudinal studies, resilience

## Abstract

Research has robustly demonstrated that children exposed to early ecological adversity are at risk for developing antisocial, externalizing behavior problems (rule breaking, aggression, disregard for others). Yet, studies have also demonstrated multifinality in developmental pathways unfolding in adversity’s aftermath, with many children showing remarkable resilience. Understanding sources of such resilience is critical, especially across different populations (Luthar et al., 2006, 2015). In Family Study (FS, 102 low-risk mothers, fathers, and infants) and Play Study (PS, 186 high-risk mother-toddler dyads), we test a model of parent–child attachment security, observed at 15 months in FS and 2.5 years in PS, as a moderator of effects of early family ecological adversity, assessed as a cumulative score of sociodemographic risks (graded for severity) at 7 months in FS and 2.5 years in PS, on children’s antisocial, externalizing problems, observed and parent-reported at 5.5 years in FS and 7 years in PS. We supported moderation for mother–child relationships in both studies: Higher early family adversity was associated with more antisocial outcomes five years later, but only for children with less secure attachments. We highlight the key role of early security as a protective factor and a source of resilience for children in families experiencing adversity.

## Introduction

Ecological perspectives (Belsky, 1984; Bronfenbrenner & Morris, 2007; Taraban & Shaw, 2018), emphasizing the importance of the family’s socioeconomic and demographic niche in children’s development, ushered in voluminous and continuously growing research on implications of early ecological adversity for children. One of the central and increasingly important questions in that research concerns factors that account for broad heterogeneity, or multifinality, in developmental pathways that unfold in the wake of early risk factors. Why are some children negatively impacted by early family adversity, but some appear remarkably resilient? This important issue, critical in developmental psychopathology, remains at the center of research on vulnerability and resilience (Cicchetti & Rogosch, 2002; Luthar & Cicchetti, 2000; Luthar et al., 2015; see also the recent Special Issue of *Development and Psychopathology*, Masten et al., 2023).

Scholarly approaches to the concept of ecological adversity have varied broadly. There is relative consensus regarding a set of several basic sociodemographic factors: parental low education and low income (Anderson et al., 2022; Baharudin & Luster, 1998; Bradley & Corwyn, 2002; Conger et al., 1994; McLoyd, 1998; Ravensbergen et al., 2025), parental young age (Berlin et al., 2002; Bornstein et al., 2006; Crugnola et al., 2014, 2019; Ragozin et al., 1982; Wakschlag et al., 2000), and number of children in the household (Keenan et al., 2007; Trentacosta et al., 2008). Beyond those, researchers have expanded the construct of ecological adversity, or risk, by including broadly ranging attributes of the family environment, such as stressful events, absence of social support, unstable family structure, quality of the neighborhood, social isolation, chaotic circumstances, interparental conflict and instability, parental psychopathology and personality, or even dysfunctional parenting, such as punishment or psychological control (e.g., Deater-Deckard et al., 1998; Evans et al., 2013; Hardi et al., 2024; Houbrechts et al., 2023). Some researchers believe that distinguishing, rather than lumping, distinct dimensions of early adversity — for example, harshness, unpredictability, threat, or deprivation — is important, because they have different implications for development (Ellis et al., 2022; McLaughlin & Sheridan, 2016). Often, researchers have distinguished between distal aspects of adversity (various attributes of the broader environment in which a family is embedded) and proximal dimensions — typically, qualities of parenting, conceptualized as mediators between the distal aspects and child developmental outcomes (J. Kim & Kochanska, 2025; Trentacosta et al., 2008). Recent research has additionally demonstrated that the effects of the distal factors, such as socioeconomic disadvantage, are distinct from the effects of proximal adversity factors, which are often interpersonal in nature (Kim-Spoon et al., 2024; Vannucci et al., 2023).

Scholars have often advocated for approaches that quantify cumulative family risk, or adversity, arguing that the combined effects of multiple risk factors can be particularly harmful (Evans et al., 2013; Rutter, 1979; Sameroff et al., 1987). However, there is substantial variation in how the measure of ecological adversity is created. A common approach is to score each of its components as present or absent and use the sum of those present as the cumulative measure of adversity (Ackerman et al., 1999; Deater-Deckard et al., 1998; Lengua et al., 2007; Rutter, 1979; Sameroff et al., 1987; Shaw et al., 1998). More sensitive or nuanced indices have also been advocated for and successfully deployed, for example, averaging standardized continuous measures of socio-demographic variables; defining a “risk threshold” for each variable and then summing those that exceed the threshold; or grading the severity of each variable and adding those graded risk scores (Belsky & Fearon, 2002; Burchinal et al., 2000, 2008; Houbrechts et al., 2023; J. Kim & Kochanska, 2025; S. Kim & Kochanska, 2021; Kochanska et al., 2007, 2012, 2013a, b).

Ecological adversity — and especially when defined broadly and cumulatively, as aforementioned — has been associated with a wide range of negative outcomes for children, including socio-emotional problems and competencies, cognitive development, neurocognitive development, and physical and mental health, to name a few (e.g., Ray et al., 2020; Scully et al., 2020; Wade et al., 2022). These findings are perhaps most consistent and robust regarding antisocial, under-regulated, or externalizing behavior problems (Appleyard et al., 2005; Blanz et al., 1991). According to an evolutionary perspective (Ellis & Del Giudice, 2019; Ellis et al., 2022; Suor et al., 2017), children in harsh, resource-deprived, uncertain environments develop such behaviors as effective strategies; aggressive, callous, or combative behaviors can promote survival and reproduction in contexts with high environmental unpredictability. Yet, although antisocial behaviors can be considered adaptive in these specific conditions, they may not be adaptive from a broader developmental and societal perspective. Aggression, defiance, rule breaking, and conflicts with and disregard for others have long-term negative effects across the immense spectrum of inter-related outcomes, such as academic performance, employment, language, social skills, peer and family relationships, legal system involvement and incarceration, and health and economic hardships (e.g., Arslan et al., 2021; Dishion & Patterson, 2006; Hyde et al., 2024, 2025; Korhonen et al., 2018; Smithers et al., 2018; Vergunst et al., 2023; Waller & Hyde, 2017).

Although evidence supporting antisocial developmental sequelae in children exposed to early family adversity is robust, research has also demonstrated considerable heterogeneity in developmental pathways triggered by adversity (Luthar et al., 2015). Children exposed to similar levels of family adversity embark on broadly diverse trajectories, with some impacted more and some showing remarkable resilience, leading to substantial multifinality in ultimate outcomes. Efforts to elucidate factors that account for such multifinality — or moderators of the impact of adversity on developmental outcomes — remain among key research enterprises in developmental psychopathology.

Attachment theory has substantially informed our understanding of origins of resilience in the wake of early adversity (Egeland et al., 1993; Masten, 2018; Sroufe, 2013). The quality of the child’s early attachment to the caregivers has been repeatedly implicated as a prominent, indeed a critical factor, with security — assessed either behaviorally (Belsky & Fearon, 2002; Cyr et al., 2014; Delker et al., 2018; Fearon & Belsky, 2011) or at the level of representation (Houbrechts et al., 2023; Madigan et al., 2016; Napoleon et al., 2023) — seen as defusing or buffering negative impacts of adversity on future cognitive, socioemotional, and behavioral outcomes, including antisocial or externalizing behavior problems.

Bowlby (1969/1982) originally conceptualized early attachment as a biobehavioral system whose key function was to assure the infant’s proximity to the caregiver, key to survival. A securely organized attachment consequently provides the child with the expectation of safe haven, the source of protection and comfort in times of stress, distress, or threat, and a secure base from which to explore in the absence of stress. Voluminous research, beyond the scope of this article, has since documented important and widely ranging positive developmental implications of such biobehavioral security, or secure attachment, and conversely, equally broad implications of insecurity, for social–emotional development. Secure attachment has often been conceptualized as an inherently cooperative and empathic developmental niche, thus promoting prosociality, altruism, and empathy (Deneault et al., 2023; Shaver et al., 2016; Stern & Cassidy, 2018), whereas insecure attachment has been reported to be associated with antisocial outcomes (Badovinac et al., 2021; DeKlyen & Greenberg, 2008; Fearon et al., 2010; Groh et al., 2017).

Further, secure attachment relationships can buffer the impact of early adversity on later externalizing behavior problems. Compared to insecure children, secure children are better able to recruit effective behavioral and biological emotion regulation strategies (Gunnar & Hostinar, 2015; Johnson et al., 2018; Perry et al., 2017), helping them cope adaptively with family stress. They are also more likely to develop positive, cooperative relationships with parents and teachers (An et al., 2021; Goffin et al., 2018), and less likely to engage in mutually coercive and adversarial dynamics that are common in parent–child dyads dealing with ecological adversities (Hyde et al., 2025). Such maladaptive dynamics often intensify and become entrenched over time, leading to antisocial problems (Dishion & Patterson, 2006).

Finally, in insecure and secure relationships, the parent and the child develop different internal representations of each other (Kochanska et al., 2019), which can substantially impact socialization processes. In the former, parents tend to view their children with hostility, and children tend to perceive their parents as untrustworthy, unavailable, mean-spirited, and ill-intentioned. In the latter, parents’ and children’s representations are positive, trusting, and benign. In turn, such representations influence parenting dynamics and child socialization outcomes. Parents with hostile representations of the child are likely to engage in harsh parenting, especially if they are also faced with ecological adversity (J. Kim & Kochanska, 2025). Children with negative representations of parents tend to resist parental control, raising the risk of externalizing problems (Kochanska & An, 2024).

Although the literature robustly and consistently supports the role of secure attachment as an important protective factor that mitigates or buffers the risk of detrimental effects of family adversity on children’s antisocial developmental path, that research is subject to three important limitations. We aim to address those in the current work.

First, the extant studies have focused mostly on children’s attachment to mothers. Research on the role of their attachment to fathers is a relative lacuna. We do not know much about how father–child security — alone and/or considered together with mother–child security as interdependent within a family system — impacts the effects of ecological adversity on development. This gap persists, despite growing emphasis on the importance of the family system approach (e.g., special issue of *Attachment and Human Development*, Cowan & Cowan, 2019; Belsky & Volling, 2014) and recently, a more specific emphasis on the role of mother- and father–child attachment configurations (Dagan et al., 2021; Dagan & Sagi-Schwartz, 2021; Kochanska & Kim, 2013).

To address this gap, in the current work, we examined how both mother–child and father–child attachment security account for the multifinality in children’s pathways to future antisocial behaviors, unfolding in the wake of early ecological family adversity. Aiming for a nuanced understanding, we modeled the family processes in two ways: examining both security with the mother and with the father, but each as an independent moderator, uniquely affecting the path from early ecological adversity to future antisocial outcomes, and additionally, testing whether the effects of the quality of attachment with one parent may be contingent on the quality of attachment with the other parent. For example, the moderating effect of attachment security with the mother on the path from ecological adversity to child antisocial outcomes can be contingent on the levels of child attachment security with the father, or vice versa. We adopted structural equation modeling that allowed us to examine the fit of the two proposed models. In addition to secure attachment, we further tested the moderating role of disorganized attachment for purely exploratory purposes, given growing evidence of disorganized attachment as a particularly significant risk (Dagan et al., 2021; Fearon & Belsky, 2011).

Second, few extant studies have examined the processes in more than one sample of families. However, the negative effects of ecological adversity and factors that exacerbate or buffer those impacts may differ in families dealing with a moderate number of stressors and families exposed to more serious and challenging situations. To explore those processes across various populations, representing a broad range of cumulative adversity, is critical for understanding mechanisms of resilience (Luthar, 2015; Luthar et al., 2006, 2015; Masten & Cicchetti, 2016).

To address this question, we utilized two studies. One was a community-based sample of 102 two-parent families of typically developing infants, not screened for any risk factors, mostly middle class, largely White, although diverse in terms of sociodemographic characteristics (Family Study, FS). We assessed ecological adversity in infancy, children’s attachment security to mothers and fathers in early toddler age, and children’s antisocial outcomes at kindergarten age.

We then aimed to replicate the tested model in a high-risk, highly stressed sample of 186 mothers and their toddlers (Play Study, PS), all of whom had low incomes and low education, and all were receiving or eligible for federal or state financial aid programs. They were ethnically and racially diverse, with no education beyond college, and many were single mothers (for details, see Kochanska et al., 2013b). Although fathers did not participate, we were able to examine processes in mother–child dyads, using constructs and measures intentionally designed to be comparable to FS: ecological adversity and child attachment at toddler age, and children’s antisocial outcomes as they began formal schooling. In both studies, we focused on the set of sociodemographic adversity factors for which there is broad consensus: parents’ education and age, family income, and number of children.

Finally, third, in the extant research, child outcomes have been measured primarily with mother reports or child self-reports. Although such measures are valid and well established, constructs solely dependent on questionnaires may be subject to limitations, such as biased answers due to social desirability, current mood, or other idiosyncratic factors (Kraemer et al., 2003; La Greca & Silverman, 1993), as well as the common method bias due to variance shared with the measures of ecological adversity, also often based on reports (Podsakoff et al., 2024). Consequently, to reduce such limitations and increase the validity and reliability of the findings in this study, we included both parent-reported and observational measures of children’s outcomes.

## Study 1: Family Study

## Method

### Participants

FS involved two-parent community families, including mothers, fathers, and their typically developing biological children. Children were born mostly in 2001. The area included a college town, a small city, and rural locations in the Midwest. Families were recruited through widely distributed advertisements, and they contacted the laboratory to volunteer to participate. They were mostly White; however, 20% of them included at least one parent who identified as non-White (see Supplement 1, Table S1 for demographic details). The University of Iowa Institutional Review Board (IRB) approved the study (Developmental Pathways to Antisocial Behavior: A Translational Research Program, 200107049). The parents signed informed consents at the beginning of the study.

Parents reported family ecological adversity when infants were 7 months (*N* = 102, 51 girls). Observational data on children’s attachment security were collected at 15 months (*N* = 101, 51 girls) and on children’s externalizing outcomes at 67 months, or 5.5 years (*N* = 92, 45 girls). At 15 months and 5.5 years, children participated in two laboratory sessions, one with each parent, led by trained female experimenters (Es). Each visit lasted approximately 2 to 4 hours and was video-recorded. The sessions included multiple structured but naturalistic paradigms and contexts. The sessions were conducted in a naturalistically furnished Living Room and a Play Room. Behavioral data were coded by multiple teams, with reliability assessed on 15 to 20% of cases, followed by regular realignments. We found no differences in ecological adversity or attachment security between families that returned at age 5.5 and those that did not return.

### Measures

#### Ecological adversity, 7 months

We created the ecological adversity index following generally the approach adopted in earlier work (J. Kim & Kochanska, 2025; Kochanska et al., 2007, 2012, 2013a). We assigned graded “adversity points” for education level and age of mothers and fathers, family income, and number of children, with higher scores denoting higher risk: parental education (for each parent) — *completed college and/or beyond* = 0, *associate’s degree* = 1, *completed high school* = 2, *did not complete high school* = 3; parental age (for each parent) — *older than 24* = 0, *23 – 24* = 1, *21 – 22* = 2, *20 years or younger* = 3; family annual income — *more than $50,000* = 0, *$40,000 – $50,000* = 1, *$30,000 – $40,000* = 2, *$20,000 – 30,000* = 3, *$10,000 – $20,000* = 4, *less than $10,000* = 5; number of children — *one or two children* = 0, *three children* = 1, *four children* = 2, *five children* = 3. For each family, all those scores were then summed into the score of demographic risk, *M* = 3.13, *SD* = 3.36, range 0 – 15.

#### Attachment security with mothers and fathers, 15 months

Trained coders completed Attachment Q-sort (AQS, Waters, 1987) for each parent–child dyad after they had viewed the laboratory session. For each dyad, the coded time was approximately 1.25 hr, encompassing parent–child interactions in multiple contexts such as free play, snack time, negotiating parental requests and prohibitions, parent “busy,” opening a gift, etc. The child also engaged with E in structured activities. Standard instructions were followed for coding and producing the final security scores. The coders sorted 90 cards describing a range of child behaviors into nine piles (10 cards each), ranging from 1 = *most uncharacteristic* to 9 = *most characteristic* of the child. Different coders were assigned to score the mother- and father–child dyads. Reliability, intra-class correlations (ICCs), ranged from .93 to .96. For each parent–child dyad, the sort was correlated with the criterion sort representing the “hypothetical most secure child,” originally provided by experts, and final attachment security scores were computed following standard instructions; security with mothers, *M* = 0.21, *SD* = 0.18, and with fathers, *M* = 0.26, *SD* = 0.19. More details are in Bendel-Stenzel et al. (2022, 2023).

#### Disorganized attachment with mothers and fathers, 15 months

Disorganized attachment was measured in the Strange Situation Procedure (SSP; Ainsworth et al., 1978), conducted as the first paradigm in the session in a room that met the required specifications, following standard guidelines. Two professional attachment coders who were blind to all other information about our participants rated the level of disorganized attachment on a scale from 1 to 9, disorganization with mothers, *M* = 2.15, *SD* = 1.97, and with fathers, *M* = 1.81, *SD* = 1.67, reliability, ICC = .87.

#### Children’s antisocial outcomes, 5.5 years

##### Violations of rules in games

Children participated in two games to win prizes. Both involved throwing objects at the targets: Bullseye — throwing Velcro balls at the target on the wall, and Ring Toss — throwing small rings at a vertical stick. E explained the rules of each game to the child and asked him or her gently but seriously to follow them and “not to cheat.” The child was left alone for 3 min. The rules made each game impossible to win (e.g., remaining in a specific place that was unrealistically far away from the target, facing away from the target, throwing each ball or ring only once). After 3 min, upon return, E “discovered” that she had given the child “wrong rules,” and the child played again, with much easier rules, until each won a prize.

Child rule-violating behavior was coded for each 3s (60 segments; kappas .95 – .99). Additionally, latencies to each violation and number of balls/rings “illegally” retrieved or manually put on target were coded (ICCs .90 – 1.00).

The standardized tallies of the violations and the (reversed) latencies were aggregated; αs were .86 and .90 for the Bullseye and Ring Toss games, respectively. The two scores correlated, *r* (91) = .67, *p* < .001, and were aggregated into one score of violations of rules, *M* = 0.00, *SD* = 0.91.

##### Moral self

E conducted a puppet interview with the child, using two puppets representing the opposite ends of each of 31 items regarding various dimensions of early conscience (e.g., rule breaking, discomfort after misbehavior, apology, etc.). For each item, one puppet presented the low end of the dimension and the other puppet — the high end (e.g., “When I break something, I try to hide it so no one finds out.”, “When I break something, I tell someone about it right away.”). The child then pointed to the puppet they felt was more like them (see J. Kim & Kochanska, 2025; Kochanska et al., 2010 for details). The child’s response to each item was coded as 0 = *choosing the low end*, as 2 = *choosing the high end*, and as 1 = *hesitating, endorsing both*. We then added all 31 items into a composite of the child’s moral self, *α* = .65, *M* = 48.09, *SD* = 7.59, and standardized it.

##### Parents’ ratings of externalizing behavior

Parents completed several well-established questionnaires. In Child Symptom Inventory–4 (CSI–4; Gadow et al., 2001; Gadow & Sprafkin, 2002; Sprafkin et al., 2002), they rated children’s behaviors from 0 = *never* to 3 = *very often*. We focused on the score of externalizing symptoms (the sum of two scales, Oppositional Defiance Disorder and Conduct Disorder); mothers, *M* = 8.33, *SD* = 5.26; fathers, *M* = 7.34, *SD* = 4.38.

In Inventory of Callous-Unemotional Traits (ICU, Frick, 2003; Frick et al., 2000; Frick & White, 2008), targeting absence of guilt and empathy, and lack of concern about rules and standards of behavior, parents rated the items from 0 = *not at all true* to 3 = *definitely true*. We computed the callous-unemotional score as the mean values of all items, mothers, *α* = .84, *M* = 0.80, *SD* = 0.32, and fathers, *α* = .86, *M* = 0.80, *SD* = 0.32.

In Health Behavior Questionnaire (HBQ; Boyce et al., 2002; Essex et al., 2002), which assesses multiple dimensions of children’s problems and competence, parents rated the items focused on the child’s overt aggression, from 1 = *never* to 3 = *often*; we then averaged the items, mothers, *α* = .64, *M* = 1.35, *SD* = 0.35, and fathers, *α* = .55, *M* = 1.33, *SD* = 0.30. Parents also rated the child’s prosocial behaviors, and we averaged those items, mothers, *α* = .88, *M* = 2.26, *SD* = 0.35; fathers, *α* = .91, *M* = 2.14, *SD* = 0.38.

Finally, we standardized and averaged all the above parental ratings (CSI-4 externalizing symptoms, ICU callous-unemotional score, HBQ overt aggression and reversed prosocial behavior) into overall mother- and father-rated externalizing behavior scores, *α* = .77, *M* = 0.00, *SD* = 0.77, and *α* = .73, *M* = 0.00, *SD* = 0.74 for mothers and fathers, respectively.

### Plan of analysis

We standardized the scores of the predictor (i.e., ecological adversity) and the moderators (i.e., attachment security with each parent) for easy interpretation of the results. We first tested the latent structure of child antisocial outcomes, encompassing violations of rules, moral self, maternal and paternal ratings of externalizing behavior, using a confirmatory factor analysis (CFA). In structural equation models, this latent variable was included as an endogenous outcome variable, and observed variables of ecological adversity and child attachment security were included as a predictor and moderators, respectively. We covaried child gender.

The data were considered missing completely at random (MCAR) according to Little’s (1988) MCAR test, χ^2^(27) = 22.16, *p* = .73. The models were estimated in Mplus 7 (Muthén & Muthén, 1998–2012) with a full information maximum likelihood estimator to handle missing data. Model fit is considered good when chi-square (χ^2^) is not significant, the comparative fit index (CFI) is larger than or equal to .95, and the root mean squared error of approximation (RMSEA) is less than or equal to .06 (Hu & Bentler, 1999).

**Figure 1.**
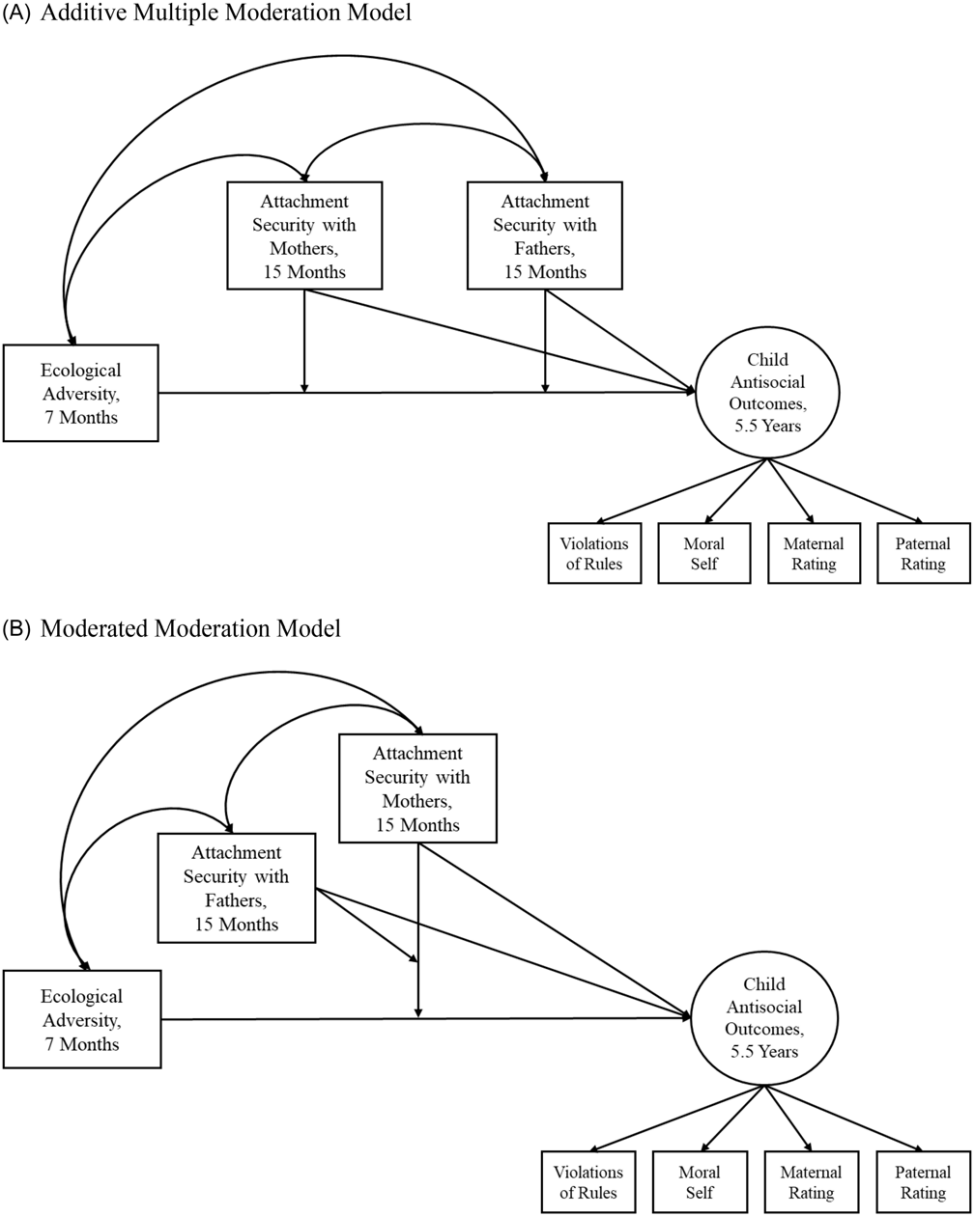
Family Study: Hypothesized models.

We compared two models. The first model (Figure 1 A) examined whether attachment security with one or both parents moderates the relations between ecological adversity and child antisocial outcomes (i.e., additive multiple moderation) by including two separate product terms of ecological adversity and child attachment security with each parent. Thus, the model tested whether the effect of ecological adversity on child antisocial outcomes depends individually and/or additively on attachment security with mothers and with fathers. The alternative model (Figure 1 B) tested whether attachment security with one parent moderates the effect of attachment security with the other parent on the association between ecological adversity and child antisocial outcome (i.e., moderated moderation or three-way interaction) by including product terms of ecological adversity, attachment security with mothers, and attachment security with fathers and of attachment security with mothers and with fathers, in addition to those included in the additive multiple moderation model. In this alternative model, the effect of ecological adversity on child antisocial outcomes is also contingent on attachment security with both mothers and fathers, but it is more nuanced as the moderation effect of attachment security with one parent can also be conditioned on attachment security to the other parent.

For significant moderation by child attachment security, simple slopes were probed and plotted at 1 standard deviation (*SD*) below and above the mean (Aiken & West, 1991) and the regions of significance (RoS) were also generated (Fraley, 2012). We also tested whether differences in child antisocial outcomes by attachment security were significant at 1 *SD* below and above the mean of ecological adversity.

## Results

### Correlations among variables

Correlations are in Table 1. In both mother- and father–child dyads, children in families with higher ecological adversity at 7 months were less securely attached at 15 months, committed more violations of rules, and were seen by their fathers as higher in externalizing conduct at age 5.5. Also in both dyads, more securely attached children committed fewer violations of rules and were seen by mothers as lower in externalizing conduct. Children more secure with mothers had higher moral selves and were seen as lower in externalizing conduct by fathers. Child attachment security and parental ratings of child externalizing conduct were significantly correlated across mother- and father–child dyads. Rule violations correlated positively with both parents’ ratings of externalizing conduct and negatively with moral self. Children with higher moral selves were seen by mothers as lower in externalizing conduct. Child disorganized attachment was not significantly related to any of the study variables in both mother–child and father–child dyads.

**Table 1.** Family Study: Correlations among variables

Variables	Ecological Adversity, 7 Months	Attachment Security, 15 months		Disorganized Attachment, 15 Months		Child Antisocial Outcomes, 5.5 Years		
		With Mothers	With Fathers	With Mothers	With Fathers	Violations of Rules	Moral Self	Maternal Rating
Attachment Security, 15 Months								
With Mothers	−.32***							
With Fathers	−.27**	.54***						
Disorganized Attachment, 15 Months								
With Mothers	−.13	−.15	−.06					
With Fathers	.06	−.20	−.08	−.13				
Child Antisocial Outcomes, 5.5 Years								
Violations of Rules	.22*	−.37***	−.22*	.06	.09			
Moral Self	−.13	.24*	.14	−.16	−.20	−.22*		
Maternal Rating	.20	−.34**	−.25*	.02	.18	.40***	−.29**	
Paternal Rating	.25*	−.29**	−.15	.12	.16	.22*	−.18	.50***

*Note.* Maternal and paternal ratings are averages of standardized scores of CSI-4 externalizing symptoms, ICU callous-unemotional traits, HBQ overt aggression, and reversed prosocial behavior. *p < .05, **p < .01, ***p < .001.

### Latent structure of child antisocial outcomes

CFA indicated that a latent variable of child antisocial outcomes with the four indicators — violations of rules, moral self, maternal and paternal ratings of externalizing behavior — fit the data well, χ^2^(2) = 0.78, *p* = .68, CFI = 1.00, RMSEA = .00, 90% confidence interval (CI) [.00, .16]. Standardized factor loadings were all significant: violations of rules .44, *p* < .001, moral self −.34, *p* < .01, maternal ratings .90, *p* < .001, and paternal ratings .55, *p* < .001.

### The effects of ecological adversity on child antisocial outcomes moderated by child attachment with mothers and fathers

**Figure 2.**
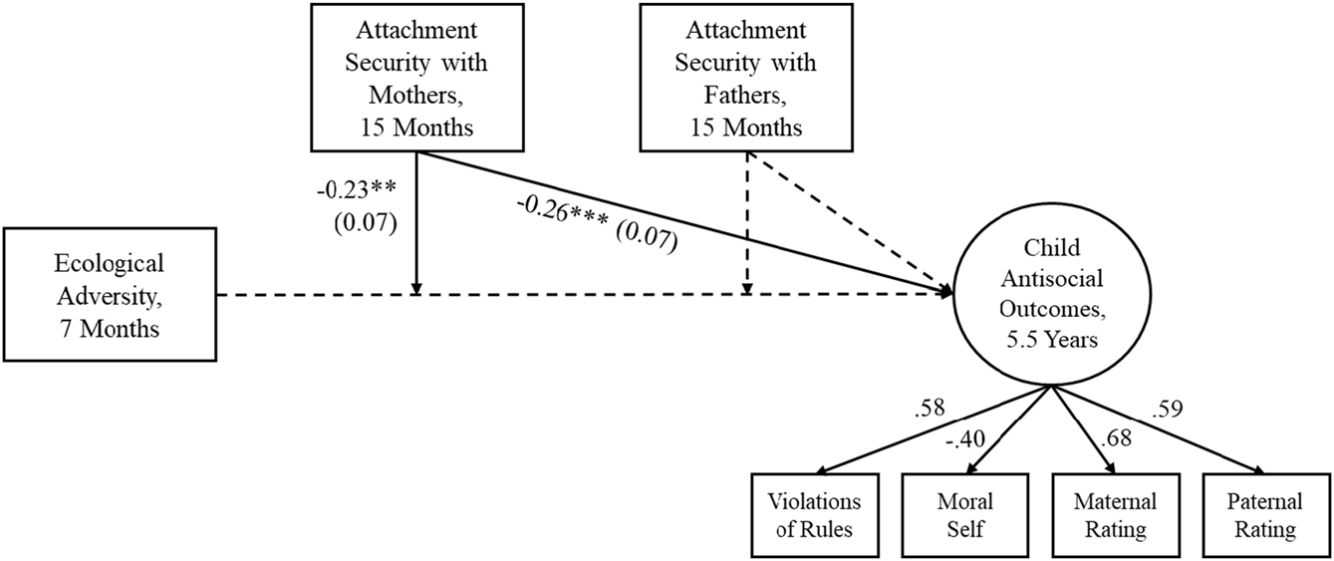
Family Study: Relations between early ecological adversity and future child antisocial outcomes moderated by attachment security with mothers and fathers. *Note*. Maternal and paternal ratings are averages of standardized scores of CSI-4 externalizing symptoms, ICU callous-unemotional traits, HBQ overt aggression, and reversed prosocial behavior. Unstandardized coefficients and standard errors in parentheses for significant paths are presented. Covariances among ecological adversity and attachment security with each parent were significant but not depicted in the figure for clarity. All factor loadings on child antisocial outcomes were standardized values and significant at *p* < .001. Child gender was included as a covariate, but is not depicted for clarity. **p < .01, ***p < .001.

We first tested the additive multiple moderation model (Figure 1 A). It had good model fit, χ^2^(26) = 29.77, *p* = .28, CFI = .97, RMSEA = .04, 90% CI [.00, .09]. The results are presented in Figure 2. Note that higher ecological adversity in infancy was related to lower attachment security with both mothers and fathers in toddlerhood, and children with more secure attachment with one parent were likely to have more secure attachment with the other parent. Those effects were included in the model but not depicted in the figure for clarity, because our focus was on the moderating roles of attachment with each parent on the path from ecological adversity to child antisocial outcomes, once the aforementioned effects were accounted for.

The effect of ecological adversity on child antisocial outcomes was not significant. More secure attachment with mothers, but not with fathers, was related to lower child antisocial outcomes. More importantly, we found a significant moderation effect of child attachment security with mothers on the relation between early ecological adversity and future child antisocial outcomes. The effect of early ecological adversity on child antisocial outcomes decreased by 0.23 for every one *SD* increase in attachment security with mothers, holding attachment security with fathers and its interaction with ecological adversity constant. Moderation by child attachment security with fathers was not significant.

**Figure 3.**
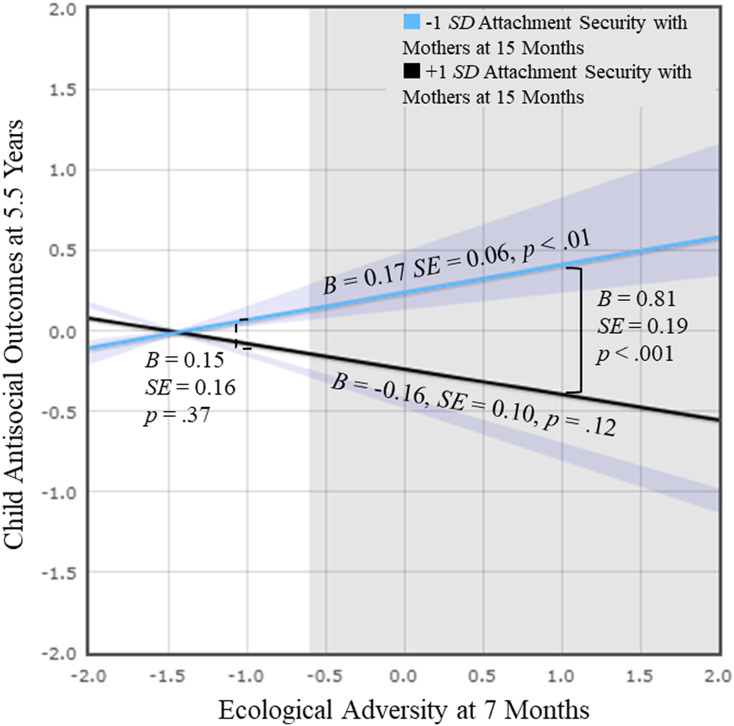
Family Study: Child attachment security with mothers moderated the relations between ecological adversity and child antisocial outcomes. *Note.* Regression based on values between 2 *SD* above and below the means of ecological adversity at 7 months and attachment security with mothers at 15 months was probed and plotted. Purple areas represent the regions of significance (RoS) with respect to attachment security with mothers (i.e., greater than 1.76 or less than −0.58). Gray areas represent RoS with respect to ecological adversity (i.e., greater than −0.60).

We tested the significance of simple slopes of attachment security with mothers at 1 *SD* below and above the mean (Figure 3). Since the moderation by attachment security with fathers was not significant, the plot was based on the model excluding it. The effect of ecological adversity on child antisocial outcomes was significant only for children who had less secure attachment with mothers, *B* = 0.17, *SE* = 0.06, *p* < .01. For those with higher attachment security with mothers, the effect was non-significant. According to the RoS testing, the effect of ecological adversity on child antisocial outcomes is significant for the values of attachment security with mothers that fall outside the regions [−0.58, 1.76]. Thus, secure attachment with mothers served as a protective factor buffering the negative influences of early ecological adversity on future child behaviors.

Differences in antisocial outcomes between children with high and low attachment security with mothers were significant only when ecological adversity was high (1 *SD* above), *B* = 0.81, *SE* = 0.19, *p* < .001. Thus, for children living in high ecological adversity, those with high attachment security with mothers showed significantly lower levels of antisocial outcomes compared to their peers with low attachment security. According to the RoS testing, the effect of attachment security with mothers on child antisocial outcomes is significant for the values of ecological adversity that fall outside the regions [−3.99, −0.60].

Next, we tested the alternative, moderated moderation model (Figure 1 B). It had less acceptable fit, χ^2^(38) = 85.70, *p* < .001, CFI = .72, RMSEA = .11, 90% CI [.08, .14], but the results from the additive multiple moderation model were replicated. The effect of ecological adversity on child antisocial outcomes was moderated by child attachment security with mothers, *B* = −0.23, *SE* = 0.07, *p* < .01, but not with fathers. We found no evidence of three-way interactions: the significant moderation by child attachment security with mothers was not contingent on child attachment security with fathers. Thus, the additive multiple moderation model was considered more parsimonious and appropriate.

### Sensitivity analyses

First, because some researchers construct ecological adversity scores by standardizing and averaging its components rather than summing individual graded scores (e.g., Belsky & Fearon, 2002; Burchinal et al., 2008; S. Kim & Kochanska, 2021), we additionally estimated an additive multiple moderation model with thus constructed scores of ecological adversity. For this model, we used the mean across standardized values of parental education levels and age, family annual income, and number of children (reversed), with a lower score indicative of higher ecological adversity. The results in the original model were replicated. The relations between early ecological adversity and future child antisocial outcomes were significant only for children with less secure attachment with mothers, *B* = −0.30, *SE* = 0.12, *p* < .01.

Second, we tested moderation in a more cumulative approach by employing a “family-level” security score (i.e., average across the standardized scores of mother–child attachment security and father–child attachment security), given the significant correlation between the two measures, *r* = .54, *p* < .001. As in the mother–child model, the relation between early ecological adversity and later child antisocial outcomes was moderated by child attachment security with parents, *B* = −0.14, *SE* = 0.06, *p* < .05. Higher ecological adversity was related to higher antisocial outcomes only for children with less secure attachment with parents, *B* = 0.20, *SE* = 0.07, *p* < .01, supporting the buffering role of high parent–child attachment security.

### Supplemental analysis: disorganized attachment as a moderator

For purely exploratory purposes, we tested moderation by child disorganized attachment with mothers and fathers measured in the SSP. As in the main analyses, the latent variable of child antisocial outcomes was included as an endogenous outcome variable, and observed variables of ecological adversity and child disorganized attachment with each parent were included as a predictor and moderators, respectively, along with product terms of ecological adversity and disorganized attachment. We covaried child gender.

We found a significant interaction effect between ecological adversity and disorganized attachment with fathers, but not with mothers, on child antisocial outcomes in an additive multiple moderation model. Higher ecological adversity was associated with higher antisocial outcomes only for children with more disorganized attachment with fathers, *B* = 0.34, *SE* = 0.07, *p* < .001.

## Study 2: Play Study

## Method

### Participants

Mothers of young children born between 2003 and 2008 volunteered for the study. Recruitment posters were broadly distributed in several counties in the Midwest, with a particular emphasis on reaching low-income families. Eligibility criteria included low income, the child’s typical developmental and health history, and the mother’s willingness to speak English while observed. The mothers were highly diverse. Demographic data are in Supplement 2, Table S2. Additional details about the recruitment procedures and the sample are in Kochanska et al. (2013b). The study was approved by the University of Iowa IRB (Play Study, 200609777). The mothers signed informed consents, and at age 7, children completed assents.

The study was designed to evaluate an early play-based intervention. The initial baseline data were collected at enrollment (average age 31 months or 2.5 years, *N* = 186, 90 girls). Ecological adversity and mother–child attachment were assessed at that time. After the baseline assessment, the dyads were randomly assigned to intervention or control groups (see Kochanska et al., 2013b). However, this article reports the data for the entire sample. We found no significant differences between the two groups in any of the outcome measures reported here, which were collected at an average age of 86 months (7 years, *N* = 118, 53 girls). Nevertheless, the intervention status was statistically controlled as a covariate in the analyses as an added safeguard.

### Measure

#### Ecological adversity, 2.5 years

We created the ecological adversity index following a similar approach to the one used in FS. We assigned the graded adversity points for the mother’s education level and age, and number of children from 0 to 3, with higher scores indicating higher risk. For family income, we calculated annual income per member of household (the annual income divided by the number of family members supported) and then assigned graded points such as *more than $7,500* = 0, *$5,000 – 7,500* = 1, *$2,500 –$5,000* = 2, *less than $2,500* = 3 (Kochanska et al., 2013a). All scores were then summed into a composite score of ecological adversity, *M* = 3.72, *SD* = 2.22, range 0 – 9.

We tested the significance of the difference in ecological adversity between FS and PS to ensure that PS families were indeed at a greater risk than FS families. To obtain parallel metrics to PS, for this analysis only, we re-scaled the family income variable in FS to be on a 0-to-3 scale as in PS, and we excluded FS father-related variables. The mean difference between FS and PS ecological adversity indices was 1.90 and significant at *p* < .001 (note that the mean of the newly created ecological adversity index for FS was 1.82, *SD* = 1.97). Thus, PS families experienced significantly higher adversity than FS families.

#### Attachment security with mothers 2.5 years

As in FS, trained coders observed the mother–child interactions (approximating 2 hours and 45 minutes) and assessed child attachment security using AQS (Waters, 1987), *M* = 0.19, *SD* = 0.20. Reliability, ICCs, ranged from .78 to .90.

#### Children’s antisocial outcomes, 7 years

##### Violations of rules in a game

The task was broadly modeled after and adapted from Eisenberg et al. (2000); for details, see Kochanska et al. (2012). The child’s task was to assemble a puzzle placed within a large box. The child was seated in a position that made it impossible to see inside the box (due to a curtain covering it). The opposite side of the box was a transparent plastic door, so the child could walk around the table and see inside the box. E asked the child to solve the puzzle only by touch — without lifting the curtain, removing any puzzle pieces from the box, or looking in from the other side. As in FS, the child was left alone for 3 min. E then returned, apologized for the wrong rules, and each child was allowed to solve the puzzle while looking at it (and all won the prize).

Coding was parallel to FS, performed for every 3s segment (rule-violating behavior, kappas .90 – .95, and latencies, ICCs all above .87). As in FS, we standardized and averaged the tallies of violations and (reversed) latencies, forming one score of violations of rules, *M* = 0.00, *SD* = 0.73, Cronbach’s *α* = .71.

##### Mothers’ ratings of externalizing behavior

We obtained parallel ratings to FS: externalizing symptoms in CSI–4, *α* = .86, *M* = 7.68, *SD* = 5.22, callous-unemotional score in ICU, *α* = .83, *M* = 0.68, *SD* = 0.32, and overt aggression, *α* = .73, *M* = 1.27, *SD* = 0.32, and prosocial behavior, *α* = .90, *M* = 2.36, *SD* = 0.35, in HBQ.

Given only one informant in PS, rather than two as in FS, we had fewer outcome measures to use to build the endogenous latent outcome variable. Consequently, we decided to include the scores from CSI-4, ICU, and HBQ overt aggression and (reversed) prosocial behavior as separate indicators, instead of aggregating them, along with the behavioral measure of rule violations, for model identification purposes.

## Results

### Correlations among variables

Correlations are in Table 2. Higher ecological adversity at 2.5 years was related to concurrent lower attachment security. Neither ecological adversity nor attachment security was related to any of the child antisocial outcomes at 7 years. Rule violations correlated with ICU, and the scores for maternal ratings of externalizing conduct showed significant intercorrelations.

**Table 2.** Play Study: Correlations among variables

Variables	Ecological Adversity, 2.5 Years	Attachment Security with Mothers, 2.5 Years	Child Antisocial Outcomes, 7 Years			
			Violations of Rules	Externalizing Symptoms	Callous Unemotional Traits	Overt Aggression
Attachment Security with Mothers, 2.5 Years	−.23**					
Child Antisocial Outcomes, 7 Years						
Violations of Rules	.11	−.11				
Externalizing Symptoms	.04	−.12	.16			
Callous Unemotional Traits	.02	.05	.19*	.51***		
Overt Aggression	.05	−.12	.11	.41***	.56***	
Prosocial Behavior	.10	.05	−.18	−.68***	−.35***	−.31***

* *p* < .05, **p < .01, ***p < .001.

### Latent structure of child antisocial outcomes

We standardized mothers’ ratings of externalizing behaviors and treated each score as an individual indicator. Model fit with five indicators (i.e., violations of rules, externalizing symptoms, callous unemotional traits, overt aggression, and reversed prosocial behavior) was not ideal, χ^2^(5) = 23.36, *p* < .001, CFI = .88, RMSEA = .18, 90% CI [.11, .25]. Thus, we included covariance between overt aggression and externalizing symptoms given their similarities and model modification indices results produced in Mplus. The model fit improved, χ^2^(4) = .90, *p* = .92, CFI = 1.00, RMSEA = .00, 90% CI [.00, .05]. Standardized factor loadings were all significant: violations of rules .21, *p* < .05, CSI-4 .53, *p* < .001, ICU .96, *p* < .001, HBQ overt aggression .43, *p* < .001, and (reversed) HBQ prosocial behavior .71, *p* < .001.

### The effects of ecological adversity on child antisocial outcomes moderated by child attachment with mothers

The data were not MCAR based on Little’s (1988) MCAR test, χ^2^(5) = 24.46, *p* < .001. Families that returned at 7 years had lower risk, *t*(184) = 4.07, *p* < .001, and higher mother–child attachment security, *t*(184) = −3.73, *p* < .001. Thus, we treated missingness following the selection modeling approach (Heckman, 1979; Little & Rubin, 2019). We included a binary variable for attrition at 7 years (0 = missing, 1 = return) and regressed it on the predictor, moderator, and outcome variables. The final model fit the data well, χ^2^(33) = 32.00, *p* = .52, CFI = 1.00, RMSEA = .00, 90% CI [.00, .05].

**Figure 4.**
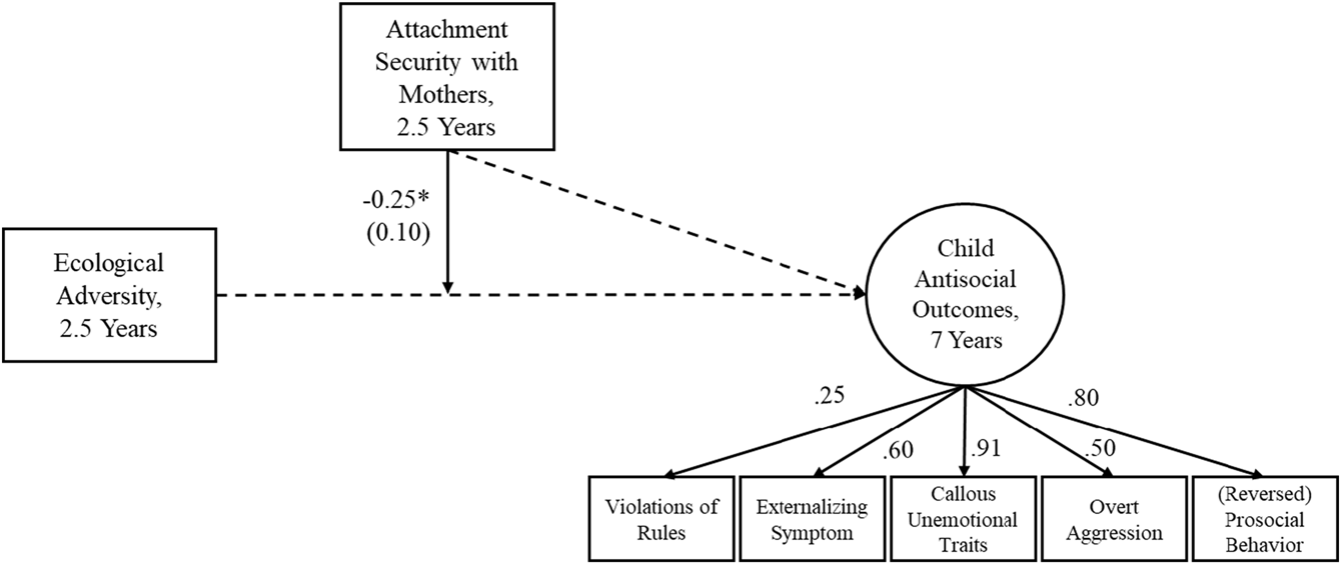
Play Study: Relations between early ecological adversity and future child antisocial outcomes moderated by attachment security with mothers. *Note*. Unstandardized coefficients and standard errors in parentheses for significant paths are presented. Covariance between ecological adversity and attachment security with mothers was significant, but is not depicted in the figure for clarity. All factor loadings on child antisocial outcomes were standardized values and significant at *ps* < .05 or .001. Covariance between externalizing symptom and overt aggression was included for model fit, but is not depicted. Child gender and intervention group were included as covariates, but are not depicted for clarity. *p < .05.

The final results are in Figure 4. Higher ecological adversity was associated with lower attachment security with mothers, but as in FS, we did not depict this for clarity. The effects of ecological adversity and attachment security with mothers on child antisocial outcomes were both not significant. However, the moderation effect of child attachment security with mothers on the relation between early ecological adversity and future child antisocial outcomes was significant. The effect of early ecological adversity on child antisocial outcomes decreased by 0.25 for every one *SD* increase in attachment security with mothers.

**Figure 5.**
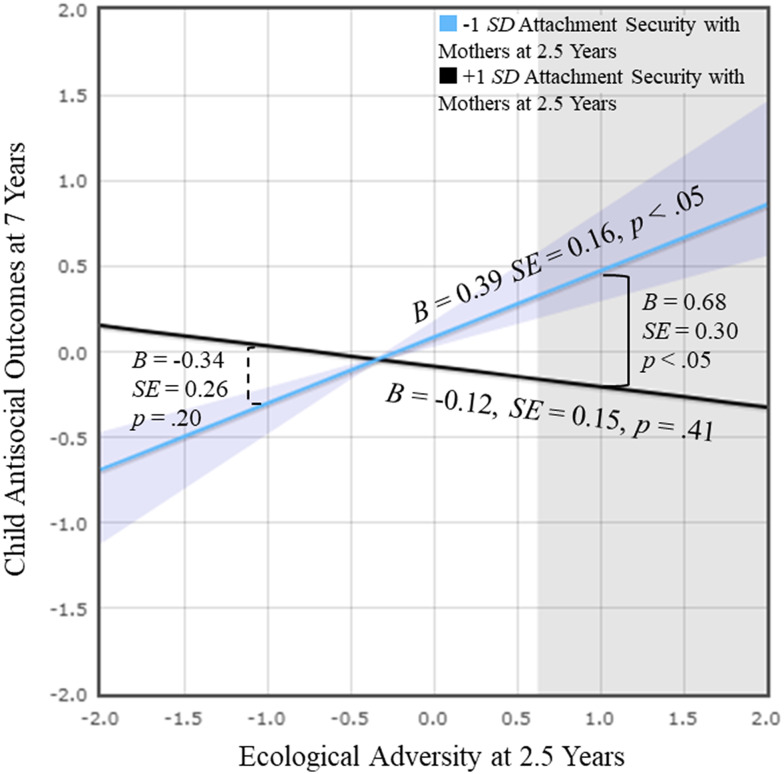
Play Study: Child attachment security with mothers moderated the relations between ecological adversity and child antisocial outcomes. *Note.* Regression based on values between 2 *SD* above and below the means of ecological adversity at 2.5 years and attachment security with mothers at 2.5 years was probed and plotted. Purple areas represent the regions of significance (RoS) with respect to attachment security with mothers (i.e., less than −0.50). Gray areas represent RoS with respect to ecological adversity (i.e., greater than 0.62).

Simple slopes were probed and plotted at 1 *SD* below and above the mean (Aiken & West, 1991; Figure 5). The effect of ecological adversity on child antisocial outcomes was significant only for children who had less secure attachment with mothers, *B* = 0.39, *SE* = 0.16, *p* < .05. For those with higher attachment security with mothers, the effect was non-significant. Thus, secure attachment with mothers buffered the negative effects of early ecological adversity on future behaviors. According to the RoS testing (Fraley, 2012), the effect of ecological adversity on child antisocial outcomes is significant for the values of attachment security with mothers that fall outside the regions [−0.50, 2.90].

As in FS, differences in antisocial outcomes between children with high and low attachment security with mothers were significant only when ecological adversity was high (1 *SD* above), *B* = 0.68, *SE* = 0.30, *p* < .05. Thus, children with high attachment security with mothers showed significantly lower levels of antisocial outcomes compared to those with low attachment security in a high-adversity context. According to the RoS testing, the effect of attachment security with mothers on child antisocial outcomes is significant for the values of ecological adversity that fall outside the regions [−2.03, 0.62].

### Sensitivity analysis

As in FS, we additionally estimated a model with the average standardized score across all indicators of ecological adversity, with a lower score denoting higher ecological adversity. The results were replicated. The relations between early ecological adversity and future child antisocial outcomes were significant only for children with less secure attachment with mothers, *B* = –0.49, *SE* = 0.17, *p* < .01.

## General discussion

Early ecological adversity can have enduring negative effects on children’s development, particularly by increasing the risk for externalizing, antisocial behavior problems. However, not all children exposed to such adversity follow maladaptive pathways, and some demonstrate notable resilience (Cicchetti & Rogosch, 2002; Luthar et al., 2015; Masten et al., 2023).

Much research has focused on elucidating factors that account for the multifinality of developmental paths unfolding in the wake of adversity. Parent–child attachment security is one well-documented protective factor (Belsky & Fearon, 2002; Cyr et al., 2014; Darling Rasmussen et al., 2019; Delker et al., 2018; Egeland et al., 1993; Fearon & Belsky, 2011; Houbrechts et al., 2023; Masten, 2018; Napoleon et al., 2023; Sroufe, 2013). Yet, prior research has predominantly focused on children’s attachment with mothers, despite the growing recognition of fathers’ unique role in child development. In addition, most studies have been limited to either low- or high-risk samples, despite calls for cross-population comparisons to better understand the mechanisms underlying resilience (Luthar et al., 2015), and despite the recent emphasis on the need for replication in social sciences, especially when interaction effects are concerned (Open Science Collaboration, 2015).

In the current work, we aimed to address these gaps by examining early attachment security with both mothers and fathers, and across both low- and high-risk families, as a factor that may buffer the detrimental impact of early ecological adversity on future antisocial behavior. Our outcome measures targeted the ages when children face important new developmental tasks and challenges following their transition to extended social contexts — kindergarten in FS and early school age in PS (Rimm-Kaufman & Pianta, 2000). Children who engage in antisocial, aggressive, externalizing behavior face cascading and often entrenched difficulties that encompass academic challenges and peer rejection (Crick & Dodge, 1994; Dishion & Patterson, 2006; Patterson et al., 1989). Consequently, understanding protective factors is an important goal.

Both studies, FS and PS, produced clear support for the protective role of mother–child attachment security. Higher ecological adversity in infancy (FS) or at toddler age (PS) was associated with more antisocial outcomes approximately 5 years later, but only for children with less secure attachment with their mothers, even after accounting for significant covariance between ecological adversity and attachment security. These findings suggest that a secure mother–child relationship serves as a protective factor that promotes children’s resilience in the face of adversity, buffering children from developing antisocial behavior across both low- and high-risk contexts. Of note, our findings in both studies indicated that the buffering effects of security are particularly significant in the context of high adversity; those findings align with evidence in the literature (Fearon & Belsky, 2011; Houbrechts et al., 2023).

How can we explain these findings? Contemporary theories of origins of antisocial behavior paint a complex biopsychosocial picture that integrates family socioeconomic factors, ecological adversity and resulting stresses, neurocognitive mechanisms, and aspects of parenting, including coercive, adversarial, and conflictual processes as all involved in elevating risks of antisocial developmental trajectories (Hyde et al., 2024, 2025). As described earlier, parent–child security can be seen as situated at the intersection of those dynamics. One, secure attachment has been shown to serve as an effective stress-buffering mechanism in general (Gunnar & Hostinar, 2015; Johnson et al., 2018; Perry et al., 2017), likely due to secure children developing more adaptive emotion regulation strategies available to be recruited and deployed under stress (Cassidy, 2021; Cassidy et al., 2025; Mikulincer & Shaver, 2019; Thompson, 2016). Two, security has also been associated with the development of prosociality and embracing parental and societal norms (An et al., 2021; Cassidy, 2021; Deneault et al., 2023; Kochanska & Kim, 2013; Shaver et al., 2016; Thompson, 2006, 2008), whereas insecurity has been robustly linked to antisocial behavior (Badovinac et al., 2021; DeKlyen & Greenberg, 2008; Fearon et al., 2010; Groh et al., 2017). Three, children securely attached to their parents are likely to develop positive mental representations of social relationships, based on their perception of parents as trustworthy, responsive, supportive, accepting, and available (Bowlby, 1969/1982; Thompson, 2021). Such representations can alter children’s subjective perceptions of stressful and adverse events (Smith & Pollak, 2021). They also foster cooperation and empathy (S. Kim & Kochanska, 2017; Shaver et al., 2016; Stern & Cassidy, 2018). Finally, secure attachment both results from and promotes positive parent–child relationships and harmonious, adaptive parenting (S. Kim et al., 2015; Kochanska et al., 2015; Thompson, 2006). Consistent with such a view of security, the findings from FS demonstrate both the main effect of mother–child security and its buffering effect, with both significant in the final equation.

In FS only, we additionally investigated whether father–child attachment security could produce similar protective effects, either independently of mother–child attachment security or in interaction with it. The moderated moderation model (i.e., whether the effect of one parent’s attachment was contingent on the other’s) was not supported. Instead, the additive moderation model indicated that mother–child and father–child attachment had independent contributions. However, the results from this additive moderation model showed that child attachment security with fathers did not have a significant main effect or moderating effect, after accounting for attachment security with mothers. This suggests that, at least at early ages, secure attachment to mothers may play a more critical role in promoting children’s resilience.

These results are somewhat inconsistent with the results of a recent meta-analysis (Dagan et al., 2021) with 1,000 individual participant data, such that no difference was found in the effects on children’s externalizing behaviors between mother–child and father–child attachment. The null findings regarding father–child attachment security may have resulted from the modest sample size of FS, which may have been insufficient to detect subtle effects of father–child attachment security. Note that we found that combined scores of mother–child and father–child attachment security had a significant moderation effect on the association between ecological adversity and child antisocial outcomes, which may suggest that father–child attachment security can play a role in mitigating the negative effect of early ecological adversity to some degree. Thus, future research with larger samples is warranted to scrutinize the degree to which father–child attachment security moderates the link between early ecological adversity and later antisocial outcomes.

Alternatively, when considering the effects of attachment security in contexts with high ecological adversity, mother–child attachment security may have greater effects than father–child relationships due to its relative focus on the safe-haven function. During early development, in particular, mothers are likely to play more extensive caregiving roles and spend more time with their children, compared to fathers (Bowlby, 1969/1982; Grossmann & Grossmann, 2019). However, this does not mean that father–child relationships are less important than mother–child relationships in long-term developmental sequelae. Father–child relationships, especially the aspect of attachment associated with secure base for exploration, may play an increasingly important role by middle childhood or adolescence as relational dynamics between parents and children change (Kerns et al., 2000; Williams & Kelly, 2005), and the child’s world expands (Paquette, 2004), as supported by a recent meta-analysis (Rodrigues et al., 2021).

This research has several limitations. First, although we included both low- and high-risk families and found consistent protective effects of mother–child attachment security, we were unable to directly compare the strength of these effects across the two groups due to differences in cohort and timing of assessments. However, it is plausible that the magnitude of the buffering effects of attachment may differ between high-risk and low-risk families. For example, the effects could be greater for high-risk families, given that the buffering effects of security are particularly significant for those with high ecological adversity in both studies and the effect sizes for the associations between insecure attachment and behavioral problems were typically larger in families with greater socioeconomic difficulties (see Badovinac et al., 2021, for a review). Future research employing group comparison is warranted to clarify these possibilities.

Second, we focused on the continuous measure of attachment security, but parent–child attachment can be approached as representing qualitatively different categories – avoidant, secure, resistant, and disorganized – and the associations between environmental risks and behavioral outcomes can differ by the quality of attachment organization (Belsky & Fearon, 2002; Groh et al., 2017; Keller et al., 2005), with disorganized attachment often seen as especially significant risk (Dagan et al., 2021; Fearon & Belsky, 2011). Of note, in our exploratory analysis in FS, in which SSP data were available, we found a significant interaction effect between ecological adversity and disorganized attachment with fathers, but not with mothers, on child antisocial outcomes. As expected, disorganized attachment with fathers amplified the negative effect of ecological adversity. This suggests the possibility of different patterns of findings if specific insecurity attachment subcategories are considered. Thus, future research including different attachment organizations as well as the level of security will provide a more comprehensive understanding of the buffering effects of attachment.

Taken together, our findings add to the literature on multifinality in developmental sequelae of early sociodemographic risk. We elucidate one important source of resilience – early parent–child attachment relationships. Although the protective role of parent–child security is known, few studies have examined its role longitudinally in the network of family relationships, including mother- and father–child dyads, in two independent samples differing in the level of adversity, deploying a multi-method approach that included a variety of behavioral observations and parental reports. Attachment security with the mother buffered children from developing antisocial outcomes, especially in a high-risk context. These results have translational implications, as they underscore the importance of enhancing parent–child attachment quality in families experiencing cumulative stresses and adversities (Berlin et al., 2016).

## Supporting information

10.1017/S0954579425100904.sm001 Kim et al. supplementary material 1 Kim et al. supplementary material

10.1017/S0954579425100904.sm002 Kim et al. supplementary material 2 Kim et al. supplementary material

## Data Availability

Although we will gladly share the coding systems or the syntax used in the analyses upon reasonable request, we are unable to share data for individual families. Our consent forms the parents signed clearly preclude any sharing of individual data, even if deidentified. The parents have consented to the data being shared in the aggregate form only, and we are ethically and legally bound to follow this agreement.

## Supplementary material

To view supplementary material for this article, please visit https://doi.org/10.1017/S0954579425100904.

## Pre-registration statement

This study was not pre-registered. When the studies began approximately two decades ago, pre-registration was much less widely implemented and rarely a concern. Although it is true that we had not pre-registered the current hypotheses and models, we have previously presented relevant findings from these data in multiple empirical and theoretical papers (many of them cited in the current paper). Additionally, of course, we specified our model and the hypotheses in great detail in our grant applications, and that information is publicly available.
