# Supplementary material for: What accounts for multifinality of the pathways from family ecological adversity to children’s future antisocial outcomes? Exploring early attachment relationships as a source of resilience in low- and high-risk samples
Source: Dev Psychopathol. 2025 Nov 13:1–16. Online ahead of print. doi: 10.1017/S0954579425100904 (PMC12825960; doi:10.1017/S0954579425100904)
Supplement: Kim et al. supplementary material 1 — Kim et al. supplementary material [file S0954579425100904sup001.docx]

What accounts for multifinality of the pathways from family ecological adversity to children’s future antisocial outcomes? Exploring early attachment relationships as a source of resilience in low- and high-risk samples

Table S1

*Family Study: Demographic characteristics of the recruited sample at entry (N = 102)*

| Characteristic | | *M* or % | | | *SD* | |
| --- | --- | --- | --- | --- | --- | --- |
| Child gender | | 50% girls | | |  | |
| Child age at entry (months) | | 7.21 | | | 0.43 | |
| Family annual income | |  | | |  | |
| Less than $10,000 | | 2.0% | | |  | |
| $10,001 – $20,000 | | 5.9% | | |  | |
| $20,001 – $30,000 | | 8.8% | | |  | |
| $30,001 – $40,000 | | 7.8% | | |  | |
| $40,001 – $50,000 | | 16.7% | | |  | |
| $50,001 – $60,000 | | 8.8% | | |  | |
| $60,001 – $70,000 | | 14.7% | | |  | |
| More than $70,001 | | 34.3% | | |  | |
| Unknown | | 1% | | |  | |
| Characteristic | Mothers | | | Fathers | | |
|  | *M* or % | | *SD* | *M* or % | | *SD* |
| Age (years) | 30.80 | | 5.30 | 32.23 | | 6.03 |
| Education |  | |  |  | |  |
| Did not complete high school | 2.9% | |  | 2.9% | |  |
| High school | 21.6% | |  | 26.5% | |  |
| Associate degree | 14.7% | |  | 17.6% | |  |
| Bachelor’s degree | 39.2% | |  | 33.3% | |  |
| Advanced degree | 20.6% | |  | 19.6% | |  |
| Unknown | 1% | |  | 0% | |  |
| Race^a^ |  | |  |  | |  |
| Asian | 1.0% | |  | 2.0% | |  |
| Black or African American | 1.0% | |  | 2.9% | |  |
| White | 91.2% | |  | 84.3% | |  |
| Pacific Islander | 1.0% | |  | 0% | |  |
| Hispanic | 2.9% | |  | 7.8% | |  |
| More than one race, other | 2.9% | |  | 2.0% | |  |
| Unknown | 0% | |  | 1% | |  |

^a^ The race categories listed were used as the official codes at the time of recruitment. “Hispanic” was a race category and not a separate ethnicity dimension.
