# Supplementary material for: What accounts for multifinality of the pathways from family ecological adversity to children’s future antisocial outcomes? Exploring early attachment relationships as a source of resilience in low- and high-risk samples
Source: Dev Psychopathol. 2025 Nov 13:1–16. Online ahead of print. doi: 10.1017/S0954579425100904 (PMC12825960; doi:10.1017/S0954579425100904)
Supplement: Kim et al. supplementary material 2 — Kim et al. supplementary material [file S0954579425100904sup002.docx]

What accounts for multifinality of the pathways from family ecological adversity to children’s future antisocial outcomes? Exploring early attachment relationships as a source of resilience in low- and high-risk samples

Table S2

*Play Study: Demographic characteristics of the recruited sample at entry (N = 186)*

| Characteristic | *M* or % | *SD* |
| --- | --- | --- |
| Child gender | 48% girls |  |
| Child age at entry (months) | 31 | 0.40 |
| Family annual income |  |  |
| Less than $10,000 | 24% |  |
| $10,001 – $20,000 | 31% |  |
| $20,001 – $30,000 | 27% |  |
| $30,001 – $40,000 | 11% |  |
| $40,001 – $50,000 | 4% |  |
| $50,001 – $60,000 | 2% |  |
| $60,001 – $70,000 | 1% |  |
| More than $70,001 | 0% |  |
| Unknown | 1% |  |
| Mothers | | |
| Characteristic | *M* or % | *SD* |
| Age (years) | 27.58 | 0.36 |
| Education |  |  |
| Did not complete high school | 5.4% |  |
| High school | 49.5% |  |
| Associate degree | 19.4% |  |
| Bachelor’s degree | 23.7% |  |
| Advanced degree | 1.6% |  |
| Unknown | 0.5% |  |
| Ethnicity |  |  |
| Hispanic/Latino | 10.8% |  |
| Not Hispanic/Latino | 87.1% |  |
| Unknown | 2.1% |  |
| Race |  |  |
| American Indian/Alaska Native | 1.6% |  |
| Asian | 1.6% |  |
| Black or African American | 14.5% |  |
| White | 72.6% |  |
| More than one race | 7.0% |  |
| Unknown | 2.7% |  |
| Relationship Status |  |  |
| Married | 54% |  |
| Cohabited with a Partner | 13% |  |
| Divorced | 6% |  |
| Single | 25% |  |
| Other | 2% |  |
